# Characterization of Turbo, a TLR Ligand-based Adjuvant for Glycoconjugate Vaccines

**DOI:** 10.4049/immunohorizons.2400040

**Published:** 2024-08-02

**Authors:** Kishore R. Alugupalli

**Affiliations:** Department of Microbiology and Immunology, Sidney Kimmel Medical College, Thomas Jefferson University, Philadelphia, PA; TurboVax Inc., Philadelphia, PA

## Abstract

Many bacterial polysaccharide vaccines, including the typhoid Vi polysaccharide (ViPS) and tetravalent meningococcal polysaccharide conjugate (MCV4) vaccines, do not incorporate adjuvants and are not highly immunogenic, particularly in infants. I found that endotoxin, a TLR4 ligand in ViPS, contributes to the immunogenicity of typhoid vaccines. Because endotoxin is pyrogenic, and its levels are highly variable in vaccines, I developed monophosphoryl lipid A, a nontoxic TLR4 ligand–based adjuvant named Turbo. Admixing Turbo with ViPS and MCV4 vaccines improved their immunogenicity across all ages and eliminated booster requirement. To understand the characteristics of this adjuvanticity, I compared Turbo with alum. Unlike alum, which polarizes the response toward the IgG1 isotype, Turbo promoted Ab class switching to all IgG isotypes with affinity maturation; the magnitude of this IgG response is durable and accompanied by the presence of long-lived plasma cells in the mouse bone marrow. In striking contrast with the pathways employed by alum, Turbo adjuvanticity is independent of NLPR3, pyroptotic cell death effector Gasdermin D, and canonical and noncanonical inflammasome activation mediated by Caspase-1 and Caspase-11, respectively. Turbo adjuvanticity is primarily dependent on the MyD88 axis and is lost in mice deficient in costimulatory molecules CD86 and CD40, indicating that Turbo adjuvanticity includes activation of these pathways. Because Turbo formulations containing either monophosphoryl lipid A or TLR2 ligands, Pam2CysSerLys4, and Pam3CysSerLys4 help generate Ab response of all IgG isotypes, as an adjuvant Turbo can improve the immunogenicity of glycoconjugate vaccines against a wide range of bacterial pathogens whose elimination requires appropriate IgG isotypes.

## Introduction

The major advantage of subunit vaccines is their safety across all ages. However, the major disadvantage, particularly for polysaccharide vaccines, is their inability to induce efficient Ab responses and long-lasting immunity across all ages. This drawback can be addressed by the addition of adjuvants, which can dramatically improve the immunogenicity of subunit vaccines by activating costimulatory pathways needed for polysaccharide vaccines (1). However, many bacterial subunit vaccines, such as typhoid Vi polysaccharide (ViPS) vaccines, the tetravalent meningococcal polysaccharide conjugate (MCV4) vaccines, and *Haemophilus influenzae* type b (Hib) vaccines, do not incorporate adjuvants (1), and the immunogenicity and efficacy of these vaccines are very low to moderate, and they require multiple boosters to induce optimal immunity in infants (1, 2). For example, Typhim Vi, an unconjugated ViPS vaccine approved by the U.S. Food and Drug Administration (FDA), induces a T cell–independent B cell response, and its efficacy is ∼55% in older children and adults, and the immunity conferred is short-lived (3). Importantly, plain ViPS vaccines do not induce Ab responses in children <2 y of age. The ViPS conjugate vaccine Typbar TCV (ViPS conjugated to tetanus toxoid), the first World Health Organization–prequalified vaccine, induces T cell–dependent Ab responses as shown in clinical trials conducted in Malawi, Bangladesh, and Nepal with an efficacy of ∼80% (4–6), but the durability of the Ab response and the long-term efficacy, particularly in infants, remains to be determined. ViPS conjugated to diphtheria toxin mutant CRM197, another typhoid conjugate vaccine, when tested in a multinational clinical trial in the Philippines, Pakistan, and India did not yield an appreciable increase in Ab titers even after two boosters (7). These clinical trials suggest a need for new adjuvant strategies for typhoid vaccines to increase their immunogenicity and efficacy in disease-endemic countries.

Despite the significant success with the pneumococcal conjugate vaccine (PCV; e.g., PREVNAR), pneumococcus continues to cause significant morbidity and mortality (8). The Centers for Disease Control and Prevention reports that in the United States alone, an estimated 900,000 illnesses, 400,000 hospitalizations, and 4000 deaths are caused by pneumococcus annually (9). In disease-endemic countries in Asia, PREVNAR did not generate an increase in Ab titers even with a booster (7), further illustrating the need for improved adjuvant strategies for PCV. Like pneumococcus and *Salmonella* Typhi, the polysaccharide-encapsulated bacterial pathogens *Neisseria meningitidis* (meningococcus) and Hib infect mostly children. Meningococcus and Hib cause lower respiratory tract infections, such as pneumonia, and many other types of serious conditions, such as meningitis, epiglottitis, cellulitis, septic arthritis, and bacteremia. Both meningococcal and Hib infections are vaccine preventable (10). Currently, two MCV4 vaccines (e.g., MENVEO and MenQuadfi) and two Hib vaccines (e.g., ActHIB and HIBERIX) are available in the United States. Like the situation with PCV, the induction of an optimal Ab response by MCV4 and Hib vaccines in infants also requires three boosters at ages 4, 6, and 12–15 mo (2, 11). Surprisingly, PREVNAR, despite having alum as an adjuvant, also requires an identical prime and multiple-booster regimen used for MCV4 and Hib vaccines, which do not incorporate adjuvants (1, 2, 11). This suggests that the utility of alum as an adjuvant is not a significant contributor in promoting the immunogenicity of PREVNAR. In fact, the immunogenicity of PREVNAR and the 23-valent unconjugated pneumococcal vaccine Pneumovax23 is dependent on the presence of TLR ligands in these vaccines (12). The multiple-booster immunization strategy used for PCV, MCV4, and Hib vaccines is not only cost prohibitive but also a reason for decreased compliance associated with boosters (10, 13), particularly for infants and children in low- and middle-income countries (14). Thus, there is a need to develop more effective adjuvant formulations to make highly immunogenic and affordable glycoconjugate vaccines for global populations.

It is known that Ags alone cannot induce an efficient Ab response (15). In fact, activation of the adaptive immune system requires the engagement of costimulatory signaling pathways in addition to primary BCR- and TCR-mediated signaling in vivo for glycoconjugate vaccines (16). Stimulation of TLR with appropriate ligands triggers the induction of costimulatory molecules, amplifies B cell activation, promotes dendritic cell maturation, and increases Ag presentation to T cells (17). TLR ligands help direct adaptive immune responses to Ags (17), and we have previously shown that a bacterial pathogen-specific Ab response requires both BCR and TLR signaling for controlling bacteremia in vivo (18, 19). Recently, I found that LPS, also known as endotoxin, a TLR4 ligand, contributes to the immunogenicity of ViPS vaccines (20). Endotoxin is a pyrogen, a highly reactogenic substance, and therefore its levels should not exceed the safety limits set by regulatory authorities. Furthermore, endotoxin levels vary from vaccine to vaccine (20) and even lot to lot (21), hence the immunogenicity mediated by its presence in vaccines is expected to be highly variable as seen with ViPS and MCV4 vaccines (20, 22). Therefore, incorporation of a defined amount of a safer TLR4 agonist-based adjuvant could make all polysaccharide vaccines immunogenic regardless of their variable levels of endogenous endotoxin content. These aspects in addition to our work (18, 20) provided a rationale for developing Turbo, a synthetic monophosphoryl lipid A (MPLA)-based adjuvant formulation to enhance the immunogenicity of bacterial polysaccharide vaccines. When adjuvanted with Turbo, typhoid vaccines and MCV4 vaccines induced significantly improved IgG responses across all ages of mice (22, 23). In this study, the mechanism of adjuvanticity of Turbo was characterized and compared with that of the widely used adjuvant alum by measuring the quantity, quality, and durability of Ag-specific IgG responses.

## Materials and Methods

### Mice

The Thomas Jefferson University Institutional Animal Care and Use Committee has approved these studies. Mice were housed in microisolator cages with free access to food and water and were maintained in a specific pathogen-free facility. Wild-type C57BL/6J (strain no. 000664), TLR4^−/−^ (strain no. 029015), MyD88^−/−^ (strain no. 009088), Trif^−/−^ (strain no. 005037), Casp1^−/−^ (032662), Casp1x11^−/−^ (strain no. 016621), IL-1R^−/−^ (strain no. 003245), NLRP3^−/−^ (strain no. 021302), GsdmD^−/−^ (strain no. 032663), CD40^−/−^ (strain no. 002928), and CD86^−/−^ (strain no. 036705) mice on C57BL/6 background were purchased from The Jackson Laboratory (Bar Harbor, ME). Age-matched mice of both sexes were used for all experiments.

### Adjuvants

The adjuvant named Turbo was prepared by mixing 1 mg of phosphorylated hexaacyldisaccharide, a synthetic MPLA, and 2 mg of 1,2-dipalmitoyl-sn-glycero-3-phosphocholine (purchased from Avanti Polar Lipids, Alabaster, AL) in chloroform. After chloroform evaporation, the contents were suspended in 1% polyethyleneglycol sorbitan monooleate (Tween 80; purchased from Sigma-Aldrich) to a concentration of 500 µg/ml MPLA and homogenized by sonication. The homogenate was extruded 50 times between two syringes connected via a 25G needle and filtered five times using a polyethersulfone membrane with pore size of 0.22 µm (Millipore) and stored at 4°C. Nanoparticle tracking analysis using NanoSight NS300 instrumentation (Malvern Instruments Ltd. Worcestershire, U.K.) revealed that the size distribution and concentration of liposomes in the adjuvant formulation was 130 ± 40 nm and 4 × 10^10^/ml, respectively. The physical characteristics and adjuvant activity were stable for at least 1 y at 4°C.

Three additional Turbo formulations were also developed by replacing MPLA with either synthetic monophosphoryl hexa-acyl lipid A, 3-deacyl (3D-6A MPLA; Avanti Polar Lipids), synthetic Pam2CysSerLys4 (Pam2CSK4), or synthetic Pam3CysSerLys4 (Pam3CSK4) purchased from InvivoGen (San Deigo, CA). To compare the adjuvanticity of Turbo with alum, I mixed Imject Alum (Thermo Scientific, Rockford, IL) with Ags and vaccines at a 1:1 ratio according to the manufacturer’s protocol.

### Antigens

4-Hydroxy-3-nitrophenylacetyl (NP) hapten conjugated to a synthetic polysaccharide Ficoll (NP-Ficoll) or chicken γ-globulins (NP-CGG) as a carrier protein are widely used model Ags to study hapten-specific T cell–independent and T cell–dependent Ab responses, respectively, in mice (24, 25). NP-Ficoll (conjugation ratio 54 NP/Ficoll) and NP-CGG (conjugation ratio 14 NP/CGG) were purchased from Biosearch Technologies (Novato, CA). Typbar TCV (ViPS conjugated to tetanus toxoid) was obtained from Bharat Biotech India Limited (Hyderabad, India). Unconjugated ViPS (lot 5, PDMI 158299) isolated from *Citrobacter freundii* strain WR7011 was obtained from the FDA (Silver Spring, MD). Phenol extraction of ViPS (lot 5, PDMI 158299) was performed as described previously (20).

### Immunization

Adult mice were immunized i.m. with Typbar TCV, unconjugated ViPS, or phenol-extracted ViPS (containing 2.5 µg of ViPS) admixed with or without Turbo (containing 5 µg of MPLA) in 50 µl volume in the right hind limb. Adult or aged mice were immunized i.m. with 50 µg of NP-CGG admixed with either Turbo (containing 5 µg MPLA) or alum in 50 µl volume. In some experiments, adult mice were reimmunized on day 28. In some experiments, Typbar TCV or NP-CGG was administered i.m. in the right hind limb, whereas Turbo was administered in the left hind limb (i.e., contralateral). Infant mice (9 d old) were immunized s.c. with 25 µg of NP-CGG admixed with or without Turbo (containing 2.5 µg of MPLA) in 25 µl volume in the scruff area between the neck and shoulder. Mice were immunized i.m. with 5 μg of NP-Ficoll admixed with or without Turbo (containing 5 µg of MPLA) in 50 µl volume. Blood samples were obtained 0, 7, 14, 21, and 28 d (or as indicated) after immunization and stored at −20°C.

### ELISA

ViPS-specific IgM, IgG, IgG1, IgG2b, IgG2c, and IgG3 were measured by coating 96-well microtiter plates (Cat. no. 442404; Nunc MaxiSorp; Invitrogen, Carlsbad, CA) with 2 µg/ml ViPS purified from *S.* Typhi clinical isolate C652464 (obtained from International Vaccine Institute, Seoul, Korea) in PBS overnight at room temperature as described previously (20). Affinity maturation of NP-specific IgG subclasses was measured by ELISA using serum samples obtained on day 21 after reimmunization as a ratio of anti-NP2 to anti-NP18 as described previously (26). The NP-specific response was measured by coating 96-well microtiter plates (Costar; Cat. no. 9017; Corning, Glendale, AZ) with NP-conjugated BSA (30, 18, or 2 NP/BSA; Biosearch Technologies, Novato, CA). Plates were washed and blocked with 1% BSA in PBS (pH 7.2; blocking buffer) for 1–2 h at room temperature. Blood from ViPS or Typbar TCV–immunized mice was diluted to 1:25 and 1:200, respectively, for IgM and IgG detection. Blood from NP-Ficoll–immunized mice was diluted to 1:100. Blood from NP-CGG–immunized mice was diluted 1:100 for infant and aged mice and 1:250 for adult mice. These dilutions were based on calibrating the linear range of detection. ViPS-specific mouse IgM, IgG, IgG1, IgG2b, IgG2c, and IgG3 levels were interpreted as ng/µl “equivalents” using normal mouse serum standards (Bethyl Laboratories, Montgomery, TX), mouse isotype-specific capture Abs, and Horseradish peroxidase conjugated anti-mouse IgM, IgG, IgG1, IgG2b, IgG2c, and IgG3 as described previously (20).

### ELISPOT

ELISPOT assays were performed as described previously (27). For NP-specific ELISPOT assays, polyvinylidene fluoride membrane-bottomed, 96-well plates (Millipore) were coated with NP30-BSA (concentration 10 μg/ml) in PBS at 4°C overnight. Plates were preincubated in RPMI 1640 supplemented with 10% FBS and HEPES (5 mM) for at least 30 min at 37°C. Bone marrow cells were then added to ELISPOT plates and incubated at 37°C in a 5% CO_2_ tissue culture incubator overnight. Cells were then washed with PBS and incubated with rat anti-mouse IgG1, IgG2b, or IgG2c or IgG3-alkaline phosphatase conjugated to alkaline phosphatase (Southern Biotechnologies). Plates were incubated for 1 h at 37°C before washing with PBS and stained with Vector blue dye according to the manufacturer’s protocol (Vector Biolabs). ELISPOT plates were then imaged and counted on an activation-induced cytidine deaminase (AID) ELISPOT reader (Advanced Imaging Devices, Strausberg, Germany).

### Statistical analysis

Data presented throughout depict pooled data from at least two independent experiments. Statistics were performed using the Prism 10 software program (GraphPad Software, La Jolla, CA), and the statistical tests are indicated in the figure legends.

## Results

### Turbo enhances Ab responses to T cell–independent and T cell–dependent Ags

**FIGURE 1.**
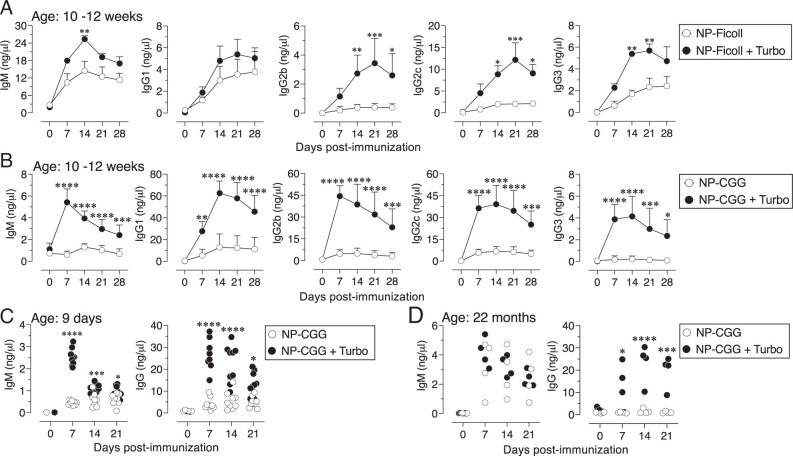
Turbo enhances Ab responses to T cell–independent and T cell–dependent Ags. Adult C57BL/6J mice (*n* = 6) 10–12 wk of age were immunized i.m. in the hind limb with (**A**) 5 µg of NP-Ficoll and (**B**) 50 µg of NP-CGG admixed with or without Turbo (5 µg of MPLA), and NP-specific IgM, IgG1, IgG2b, IgG2c, and IgG3 levels were measured by ELISA. (**C**) Infant C57BL/6J mice (9 d old) were immunized s.c. with 25 µg of NP-CGG admixed with or without Turbo adjuvant (containing 2.5 µg of MPLA) in 25 µl volume in the scruff area between neck and shoulder. (**D**) Aged C57BL/6J mice (22 mo) were immunized with 50 µg of NP-CGG admixed with or without Turbo adjuvant i.m. in the thigh of the hind limb with 50 µl of volume. NP-specific IgM and IgG levels were measured by ELISA. Statistics were done using two-way ANOVA Sidak’s multiple comparisons test. Each dot represents an individual mouse, and statistically significant differences were indicated as ****p < 0.0001, ***p < 0.001, **p < 0.01, *p < 0.05.

NP-Ficoll is a well-studied synthetic Ag (24), which induces T cell–independent Ab response even in mice deficient in MyD88, an adaptor protein required for signaling through all TLR members except TLR3 (28). The adjuvanticity of Turbo, a TLR4 ligand-based formulation, therefore was tested using NP-Ficoll. I found that admixing Turbo with NP-Ficoll significantly increased NP-specific IgM, IgG2b, IgG2c, and IgG3 responses in mice (Fig. 1A). Studying immune responses using hapten conjugated to carrier proteins led to the invention of bacterial polysaccharide-conjugate vaccines (29–31). To test the adjuvanticity of Turbo in the context of T cell–dependent Ag, I chose a well-studied non–bacterial-derived haptenated Ag NP-CGG (25). When NP-CGG was admixed with Turbo, a dramatically improved NP-specific IgM, IgG1, IgG2b, IgG2c, and IgG3 isotype response in adult mice was observed (Fig. 1B). These data indicate that Turbo can serve as an adjuvant for T cell–independent and T cell–dependent Ags such as unconjugated or conjugated polysaccharide vaccines, respectively.

The lack of an efficient Ab response, particularly to polysaccharide Ags, in human infants and infant mice is due to several factors, including incomplete B cell development and restricted BCR repertoire (32–34). Aged individuals also do not respond efficiently to vaccines because of immune senescence (35). Studies in mice have shown that the impairment in immune responses in aged mice is due to several parameters, including fewer APCs and their reduced interaction with T cells (36). However, when NP-CGG was adjuvanted with Turbo, both infant and aged mice showed a significant improvement in IgG responses (Fig. 1C, 1D), indicating that Turbo can serve as an adjuvant across all ages.

### Turbo adjuvanticity is quantitatively and qualitatively distinct from that provided by alum

**FIGURE 2.**
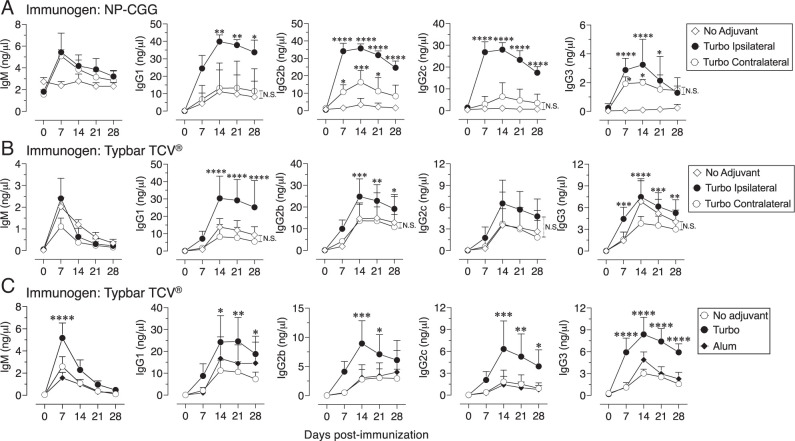
Admixing Turbo with Ags is required for its adjuvanticity. (**A**) 50 µg of NP-CGG (*n* = 4) or (**B**) Typbar TCV vaccine containing 2.5 µg of ViPS (*n* = 12) given alone or admixed with Turbo (5 µg of MPLA) was administered i.m. with (A) in the right thigh of the hind limb (ipsilateral) of 10- to 12-wk-old C57BL6/J mice. A group of mice that were immunized with NP-CGG or Typbar TCV alone also received Turbo in the left thigh of the hind limb (contralateral). NP-specific or ViPS-specific IgM, IgG1, IgG2b, IgG2c, and IgG3 levels were measured by ELISA. (**C**) Typbar TCV vaccine containing 2.5 µg of ViPS given alone or admixed with either Turbo (5 µg of MPLA) or alum (Imject Alum; 1 mg of aluminum hydroxide and 1 mg of magnesium hydroxide) and immunized i.m. measured Ab responses. Statistics were done using two-way ANOVA Sidak’s multiple comparisons test, and statistically significant differences were indicated as ****p < 0.0001, ***p < 0.001, **p < 0.001, *p < 0.05. N.S., not significant.

In nonhuman primates, efficient germinal center and Ab responses occur when an eOD-GT8 60-mer nanoparticle vaccine adjuvanted with ISCOMATRIX is administered ipsilaterally, but not contralaterally (37). Therefore, NP-CGG or Typbar TCV admixed with Turbo is expected to induce an efficient Ab response, but not when Turbo is administered separately. Indeed, I found that with two distinct Ag systems, ipsilateral administration of Turbo, but not contralateral administration (left thigh), relative to the Ag injection site (right thigh) induced robust anti-NP IgG1, IgG2b, IgG2c, and IgG3 responses (Fig. 2A) and anti-ViPS IgG1, IgG2b, and IgG3 responses (Fig. 2B). These data demonstrate that the Ag and adjuvant need to be at the same site of injection, and therefore admixing Turbo with an Ag/vaccine is appropriate for studying Turbo adjuvanticity.

Most bacterial polysaccharide vaccines do not incorporate adjuvants (1), and their immunogenicity is variable in both mice and humans. Alum is the most widely used adjuvants in many different vaccines, and to date, it is the only adjuvant incorporated into a small number of bacterial polysaccharide conjugate vaccines (e.g., PREVNAR). Therefore, I compared the adjuvanticity induced by Turbo with that of alum using Typbar TCV as the Ag. I found that Turbo induces a significantly higher anti-ViPS IgG1, IgG2b, IgG2c, and IgG3 response compared with alum (Fig. 2C).

**FIGURE 3.**
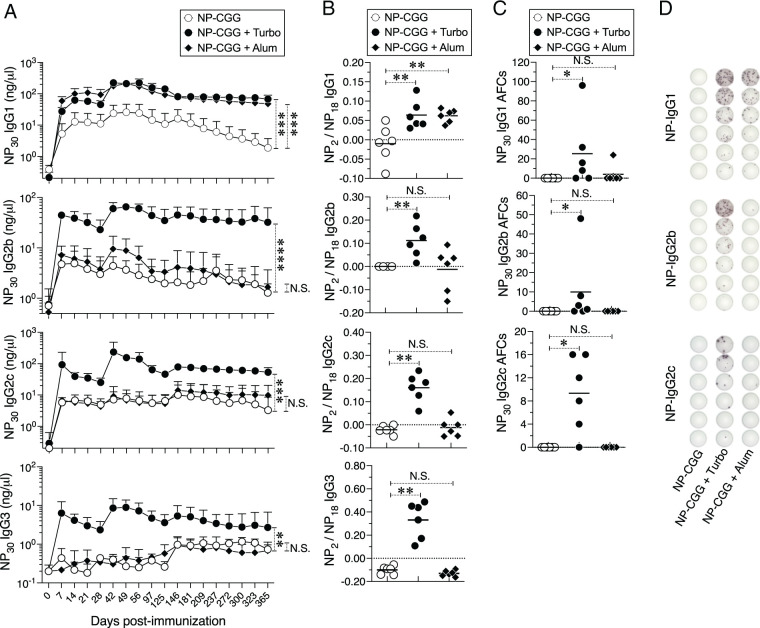
Comparison of the adjuvanticity of Turbo with that of alum. Male and female C57BL6/J mice (*n* = 6) were immunized i.m. in the thigh of the hind limb with NP-CGG, admixed with Turbo (5 µg of MPLA) or Alum (Imject Alum; 1 mg of aluminum hydroxide and 1 mg of magnesium hydroxide). On day 28, mice were boosted with the same Ag and adjuvant combinations. NP-specific Ab levels were measured by ELISA. The ELISA values obtained with NP_30_-BSA–, NP_18_-BSA–, or NP_2_-BSA–coated plates measure NP-specific Abs of low to high affinities, respectively. (**A**) Turbo-adjuvanted Ag shows durable IgG responses of all four isotypes as shown with mean ± SD. Statistical analyses were done using two-way ANOVA Sidak’s multiple comparisons test, and statistically significant differences were indicated as ****p < 0.0001, ***p < 0.001, **p < 0.001, *p < 0.05. (**B**) Turbo promotes affinity maturation of all four IgG subclasses measured by ELISA using serum samples obtained on day 21 after reimmunization as a ratio of anti-NP_2_ to anti-NP_18_ IgG1, IgG2b, IgG2c, and IgG3. Each dot represents an individual mouse, and the black bar represents mean. Statistical differences were determined by Mann–Whitney *U* test. (**C**) AFCs producing anti-NP IgG1, IgG2b, and IgG2c were enumerated by ELISpot assay. The number of AFCs per one million bone marrow cells was shown. Each dot represents an individual mouse, and the black bar represents mean. Statistical differences were determined by Mann–Whitney *U* test. (**D**) Representative well images of the ELISPOT assay for each group are shown at various (2-fold) dilutions. N.S., not significant.

I have previously found that associated TLR4 ligands such as endotoxin contribute to the immunogenicity of Typbar TCV (20). NP-CGG is not a bacterially derived Ag, and therefore is not expected to contain endotoxin or TLR ligands. As such, NP-CGG induces a poor response without adjuvant. Therefore, I compared the effect of Turbo with alum using NP-CGG as the immunogen. As expected, alum, which is known to polarize toward a Th2 response, promoted the anti-NP IgG1 response (Fig. 3A). In striking contrast, Turbo promoted not only the anti-NP IgG1 response but also improved anti-NP IgG2b, IgG2c, and IgG3 responses (Fig. 3A), suggesting there is a lack of a bias toward Th1 or Th2 when Turbo is used as an adjuvant. Furthermore, when Turbo was incorporated in the booster immunization, an increase in the levels of all four IgG isotypes with an increase in affinity maturation of all four IgG isotypes was observed (Fig. 3B). This suggests that Turbo promotes quantitatively and qualitatively distinct germinal center responses compared with alum. Furthermore, Turbo as an adjuvant promoted sustained levels of NP-specific Abs of all four IgG isotypes for at least 1 y in mice, whereas alum promoted only IgG1 (Fig. 3A). ELISPOT analysis of bone marrow cells showed that NP-specific IgG1, IgG2b, and IgG2c Ab-forming cells (AFCs) are detectible even after 1 y in mice immunized with Turbo as an adjuvant (Fig. 3C, 3D). Unlike the other IgG isotypes, IgG3 levels were an order of magnitude lower, and possibly because of the detection limit of the ELISPOT assay, NP-specific IgG3 AFCs were not observed with any immunization. These data indicate that Turbo promotes the development of long-lived plasma cells of various IgG isotypes.

### Turbo adjuvanticity primarily requires TLR4 and MyD88, but not those pathways activated by alum

**FIGURE 4.**
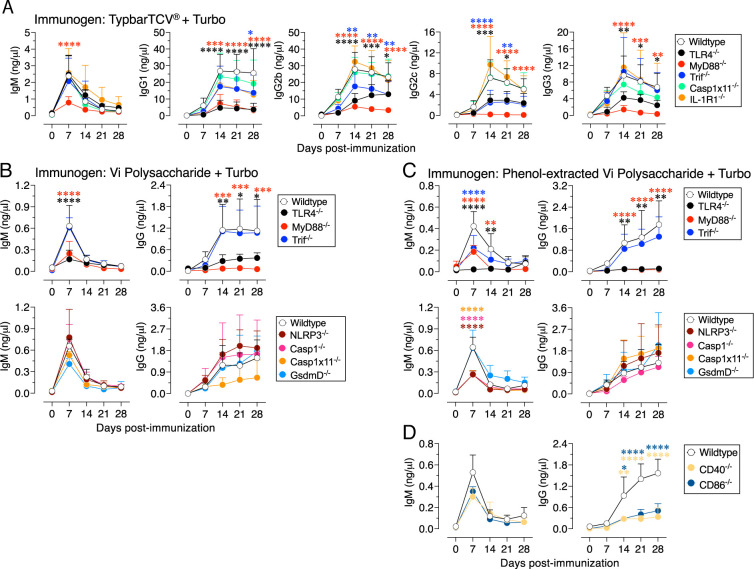
Turbo adjuvanticity requires TLR4-MyD88 axis, as well as costimulatory molecules CD40 and CD86. Wild-type (C57BL/6J) or indicated gene-deficient male and female mice (*n* = 5–8) 8–10 wk of age were immunized i.m. with 2.5 µg of (**A**) Typbar TCV, (**B**) ViPS, or (**C** and **D**) phenol-extracted ViPS admixed with Turbo (5 µg of MPLA). ViPS-specific IgM and IgG levels were measured by ELISA. Statistics were done using two-way ANOVA Sidak’s multiple comparisons test, and statistically significant differences were indicated as ****p < 0.0001, ***p < 0.001, **p < 0.001, *p < 0.05.

Alum activates the NLRP3 inflammasome, Caspase-1, and maturation of IL-1β and the pyroptotic cell death effector Gasdermin D (38, 39). In contrast, IL-1R and canonical or noncanonical inflammasome activation mediated by Caspase-1 or Caspase-11, respectively, are not required for Turbo adjuvanticity when Typbar TCV is used as the immunogen (Fig. 4A). Alum does not induce signaling via TLR4 or MyD88 (28, 40). In contrast, TLR4 and MyD88 deficiency affected all four IgG isotypes when Turbo was used as an adjuvant (Fig. 4A). The partial impairment of IgG2b and IgG2c responses in Trif^−/−^ mice suggests that both MyD88 and Trif axes are required for IgG2b and IgG2c. These data demonstrate that Turbo adjuvanticity is distinct from that of alum, and that the TLR4-MyD88 axis plays a major role, whereas the TLR4-Trif axis plays a minor role in Turbo-induced adjuvanticity to Typbar TCV, a T cell–dependent Ag.

To test whether similar types of TLR4 signaling axes are required for T cell–independent responses, I tested Turbo adjuvanticity with unconjugated ViPS. With this Ag I found that mice deficient in TLR4 and MyD88, but not Trif, showed a severe impairment in the anti-ViPS IgM and IgG responses, indicating that the TLR4-MyD88 axis is critical for Turbo-driven adjuvanticity to T cell–independent Ab responses as well (Fig. 4B). As expected, neither IgM nor IgG responses to Turbo-adjuvanted ViPS were affected in mice deficient in NLRP3, Caspase-1, Caspase-11, or Gasdermin D (Fig. 4B).

ViPS vaccines and preparations contain endogenous TLR4 ligands that contribute to anti-ViPS immunogenicity (20). I have previously shown that phenol extraction eliminates TLR4 ligand activity in ViPS preparations, as well as their immunogenicity (20). To confirm Turbo-driven adjuvanticity in the absence of any endogenous TLR4 ligand activity in ViPS, I immunized mice with phenol-extracted ViPS admixed with Turbo. Even in this Ag combination Turbo adjuvanticity was dependent on the TLR4-MyD88 axis, but not Trif, for generating anti-ViPS IgM and IgG (Fig. 4C). As seen with native unconjugated ViPS (Fig. 4B), mice deficient in NLRP3, Caspase-1, Caspase-11, or Gasdermin D, when immunized with Turbo-adjuvanted phenol-extracted ViPS, generated anti-ViPS IgG responses that were comparable with wild-type mice (Fig. 4C). In conclusion, use of all three ViPS Ag systems (Fig. 4A–C) demonstrates that Turbo activity is not only distinct from that of alum but also indicates that the TLR4-MyD88 axis is the primary signaling mechanism leading to Turbo-mediated adjuvanticity.

### Turbo adjuvanticity is dependent on the costimulatory molecules CD86 and CD40

An efficient B cell response requires the engagement of costimulatory molecules in addition to BCR signaling (15). Turbo upregulates costimulatory molecules CD86 and CD40 on murine B cells (22). When phenol-extracted ViPS (a preparation devoid of endogenous TLR4 ligands) (20) admixed with Turbo was used as an immunogen, mice deficient in CD86 or CD40 were impaired in generating anti-ViPS IgG responses (Fig. 4D). These results are consistent with the notion that in addition to BCR signaling, costimulatory pathways provided by CD86 and CD40 are required for the immunogenicity of not only polysaccharide conjugate vaccines (16, 22) but also for the immunogenicity of unconjugated polysaccharide vaccines (Fig. 4D). Thus, Turbo fulfills the crucial adjuvant requirement for making efficient IgG responses.

### Turbo formulation containing TLR2 ligands also promotes Ab responses to typhoid glycoconjugate vaccine

**FIGURE 5.**
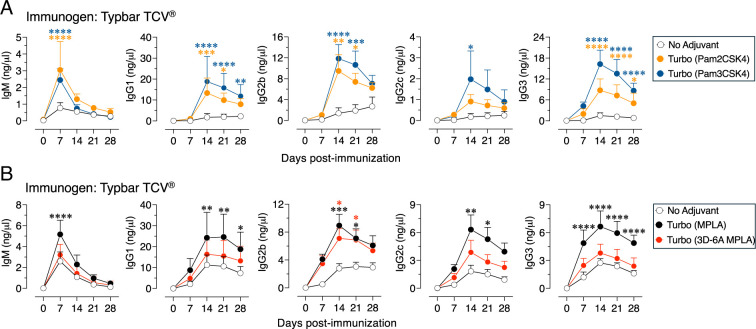
Immunogenicity promoted by Turbo formulations containing various TLR2 and TLR4 ligands. Wild-type (C57BL/6J) male and female mice (*n* = 6) 8 wk of age were immunized i.m. with 2.5 µg of Typbar TCV (**A**) admixed with Turbo formulation containing 5 µg of Pam2CSK4 or Pam3CSK4, or (**B**) admixed with Turbo formulation containing 5 µg of MPLA or 3D-6A MPLA. ViPS-specific IgM and IgG isotype levels were measured by ELISA. Statistics were done using two-way ANOVA Sidak’s multiple comparisons test, and statistically significant differences were indicated as ****p < 0.0001, ***p < 0.001, **p < 0.001, *p < 0.05.

Because MPLA-based Turbo adjuvanticity is primarily dependent on MyD88 (Fig. 4), formulations containing Pam2CSK4, an agonist for TLR2/6 heterodimer, or Pam3CSK4, an agonist for TLR2/1 heterodimer, which also signal via MyD88, were tested. I found that both formulations significantly enhanced all isotypes when admixed with Typbar TCV (Fig. 5A). Compared with MPLA, the 3D-6A MPLA is >10-fold weaker than TLR4 agonist (41), and the adjuvanticity mediated by 3D-6A MPLA-based Turbo formulation is not as efficient as that mediated by MPLA (Fig. 5B).

## Discussion

In polysaccharide protein conjugate vaccines, the polysaccharide and peptide moieties are specific Ags for BCR and TCR, respectively (1, 2). Although BCR and TCR activation are the primary signals for B and T cells, a second signal provided by adjuvants is an important requirement for efficient B and T cell responses (42, 43). For example, the Ab response to a hapten-conjugated Ag such as NP-CGG, which contains epitopes for both the BCR and TCR, does not occur efficiently in the absence of adjuvant as seen in Fig. 3A. Therefore, conjugation alone is not sufficient for the immunogenicity of polysaccharide conjugate vaccines. In support of this, conjugate vaccines containing antigenic epitopes for the BCR and TCR (e.g., MCV4) without adjuvants are not expected to initiate an efficient response. In fact, for the induction of an optimal Ab response in infants, many polysaccharide vaccines, including MCV4, require a first immunization at 2 mo of age and three boosters at ages 4, 6, and 12–15 mo (2, 11). However, admixing Turbo with MCV4 vaccines not only promotes the immunogenicity of all four serogroup polysaccharides in the MCV4 across all ages but also minimizes the requirement for booster immunizations (22), indicating that the second signal generated by the adjuvant plays a crucial role in the immunogenicity of bacterial polysaccharide conjugate vaccines.

The immunogenicity of Group B *Streptococcus* type III polysaccharide conjugated to tetanus toxoid adjuvanted with alum requires CD80, CD86, CD40, and MHC class II in vivo (16). I recently showed that Turbo induces the upregulation of the costimulatory molecules CD86, CD40, and MHC class II, but not CD80, on B cells (22). Mice deficient in CD86, despite having normal CD80 (44), did not generate an IgG response either to ViPS (Fig. 4D) or to MCV4 vaccines when adjuvanted with Turbo (22). These data indicate that unlike the scenario with alum-adjuvanted Group B *Streptococcus* type III polysaccharide conjugated to tetanus toxoid, where both CD80 and CD86 are required (16), the CD80 is not a part of the mechanism by which Turbo acts as an adjuvant. CD40 is known to upregulate the expression of AID in germinal center B cells, which is required for Ig class switching and affinity maturation (45). AID is also induced by BCR and TLR stimulation even in the absence of T cell help (46). Although CD40 expression induced by Turbo (22) or LPS alone is low on B cells stimulated in vitro (47), CD40^−/−^ mice, when immunized with Turbo-admixed ViPS, failed to generate an efficient IgG response (Fig. 4D) similar to that seen in CD40^−/−^ mice immunized with Turbo-adjuvanted MCV4 (22). The reduced Turbo adjuvanticity in CD86^−/−^ mice suggests that CD86 serves as the costimulatory molecule in the initial phase of B and T cell activation, whereas CD40 signaling plays an important role in the downstream phase of germinal center or extrafollicular B cell activation in the context of a T cell–dependent Ag (e.g., MCV4) (22) and T cell–independent Ag (e.g., ViPS) (Fig. 4D), respectively. Interestingly, the short-lived IgM response to ViPS (Fig. 4D) and MCV4 (22), which is induced primarily by BCR crosslinking by polysaccharides containing repetitive epitopes, is comparable in wild-type and CD40^−/−^ or CD86^−/−^ mice, unlike IgG responses. Therefore, adjuvants like Turbo are expected to promote efficient IgG Ab responses to both unconjugated and conjugated polysaccharide vaccines by engaging CD86 and CD40.

MPLA is structurally like lipid A, the active moiety of LPS/endotoxin, but lacks one phosphate group and does not have the inflammatory profiles or endotoxic activity of lipid A (41). Although both lipid A and MPLA signal through TLR4, MPLA-driven signaling is qualitatively and quantitatively distinct from that of LPS based on differences in CD14/TLR4/MD2 interaction characteristics, as well as the kinetics of signaling between LPS and MPLA (48, 49). Furthermore, MPLA does not promote LPS-induced tolerance to the same extent as that induced by LPS (50). Indeed, despite containing LPS/endotoxin, ViPS and Typbar TCV generated a robust response when adjuvanted with Turbo (20), and this was dependent on TLR4 and MyD88 (Fig. 4A–C). This suggests that unlike MPLA dissolved in dimethyl sulfoxide, which is a commonly used solvent for in vitro assays (because MPLA is not soluble in water), where it signals mainly via the TLR4-Trif axis in primary cells and cell lines for cytokine production (51), the MPLA in the Turbo formulation is primarily dependent on the TLR4-MyD88 axis in vivo for Ag-specific IgG responses (Fig. 4A–C).

PREVNAR, despite having alum as an adjuvant, requires an identical prime and multiple-booster regimen used for MCV4 and Hib vaccines (2, 8). This suggests that alum is not a significant contributor in promoting the immunogenicity of PREVNAR. A direct comparison of the adjuvanticity of alum with that of Turbo in the context of two distinct T cell–dependent Ags, Typbar TCV (Fig. 2C) and NP-CGG (Fig. 3A), showed that Turbo improves production of all IgG isotypes with affinity maturation (Fig. 3B). This suggests that a wide range of complement and IgG Fc receptor–mediated protective mechanisms would be promoted using Turbo as an adjuvant. *Salmonella* serovars have distinct pathogenesis profiles; they likely require different Ig isotypes for their control. In fact, the control of *S. pneumoniae* and *N. meningitidis* requires opsonophagocytic and bactericidal Abs, respectively, illustrating the need for an adjuvant to induce multiple Ig isotypes, because each Ig isotype has a specialized function and unique distribution (52). For example, IgG2 is efficient in opsonophagocytosis, whereas IgG1 and IgG3 are efficient in mediating complement-dependent bactericidal activity (52–54). Because Turbo promotes class switching to all four IgG isotypes, Turbo as an adjuvant will enable polyfunctional Abs against a wide range of bacterial pathogens. The data from Fig. 2A and 2B demonstrate that the Ag/vaccine and Turbo need to be at the same site, suggesting that Turbo can be premixed or bedside mixed with any glycoconjugate vaccines. Therefore, admixing Turbo into FDA-approved or World Health Organization–prequalified monovalent and multivalent vaccines (e.g., typhoid, Hib, MCV4, and PCV), as well as those glycoconjugate vaccines against Group A *Streptococcus* (55), *Escherichia coli* (56), *Shigella* (57), and nontyphoidal *Salmonella* (58) that are being developed, is expected to improve the immunogenicity and provide durable protection from these bacterial pathogens. By sparing the Ag dose when adjuvanted with Turbo as shown with ViPS (23) and eliminating booster requirement as shown with MCV4 (22), Turbo-adjuvanted vaccines can be cost-effective and increase the compliance associated with minimizing boosters, particularly for individuals in low- and middle-income countries across all ages.
